# Report of the Committee on Suits for Mal-Practice

**Published:** 1859-06

**Authors:** C. H Spilman

**Affiliations:** Harrodsburg, Ky.


*Report of the Committee on Suits for Mal-Practice.—Submitted at the Fourth Annual Meeting of the Kentucky Medical Society.*
By C. H SPILMAN, M. D., of Harrodsburg, Ky.

MR. PRESIDENT:—The subject selected for the present hour, which it becomes my duty to present for your considerations, is manifestly complicated, involved, and intricate I very much fear, therefore, that I shall not be able to answer the expectations of our Society, whose paramount obligation it has ever been, and whose ardent desire it will ever continue to be, not only to maintain the honor and usefulness of our profession, but, as far as practicable, to secure the safety of the public, and protect it from imposition.

The task imposed on me may prove an ungracious one; but I have no apology to offer, nor shall I shrink from the responsibilty. It is as difficult as ungracious. A mere glance at the subject must convince any one that truth does not lie upon the surface. It is a controvertible subject. So unsettled and indeterminate the premises, and so constant the alternation of lights and shadows in the field of medico-legal science that the conclusions attainable by the most logical course of reasoning of which the subject is susceptible in the present state of our knowledge, are necsssarily, to some extent, inconclusive and unsatisfactory. I am free to admit, that some of the positions I have taken, I approached with hesitation, not being fully satisfied myself of their correctness. I can not hope, therefore to explore the entire field before me, without, at times, occupying disputed territory. I lay it as an offering, however, upon the altar, not entirely without the hope that it may be an acceptable one; and whilst it must needs undergo the ordeal criticism, and it may be, of animadversion, I feel a comforting assurance of being invested with the mantle of charity, as a shield against the shafts of bitter invective, or unnecessary rigor in the examination of the errors with which it may very probably abound.

It has been remarked by one of the soundest, most practical modern philosophers, that the learned professions of law, medicine, and theology, constitute the three grand pillars of our social compact. It is only, however, where there is a full recognition on the part of the members of these professions respectively, of the responsibilities which their positions impose, the ennobling truths which they are commissioned to bear and the sacred trusts confided to their hand that this sentiment can command our unqualified assent. Without that pre-eminence of intellectual culture, and superior excellence of moral character, which ought to be implied in the very positions they occupy, ther influence, instead of elevating and ameliorating the general condition of society, would tend, most irresistibly, to its intellectual degradation and moral corruption.

Whatever may have been the fortunes of the medical profession in the ever-varying phases of society, at different periods of the world's history, from its origin to its present high state of improvement and usefulness, there is one consideration eminently calculated to inspire its cultivator with a just pride, and a profound sense of the interesting and important relations it sustains to society, and the weighty responsibilities those relations impose.

The high appreciation of the healing art, and the honor bestowed on it, amounting almost to veneration, from the earliest ages of antiquity, afford a lofty idea of the estimation in which it was held—the high expectations of the public, and the important character of the mission with which it is invested.

A faint conception of the honor paid to medicine at an early period of our history, may be gathered from the sacred writings, from which we extract the following beautifully simple examples:

"Honor a physician with the honor due unto him, for the uses ye may have of him, for the Lord hath created him.

"For the Most High cometh healing, and he shall receive honor of the king.

"The skill of the physician shall lift up his head, and in the sight of great men he shall be in admiration.

"The lord hath created medicines out of the earth, and he that is wise will not abhor them.

"With such doth he heal them, and taketh away their pains."

If there be not a manifest impropriety in applying immortality to man without reference to a future state of existence, then, certainly, medicine has some reason to be proud of the immortality conferred on some of its early cultivators. Without going into fabulous or poetic regions, in which some of the early physicians are represented as receiving not only the highest honors, but the homage of the multitude, the high estimation in which some of the productions of the pioneers of the profession are still held, afford to their successors, who distinguish themselves in the healing art, a guarantee of a future renown, if not as brilliant, no less solid and enduring than theirs.

It is perhaps true, that the pathway to distinction is more difficult now than formerly,—that to acquire eminence in the profession, higher attainments, and a larger measure of knowledge and skill, are requisite.

The opinions and doctrines of one man may never again sway the the entire medical word, as did those of the Coan sage. The great thirst for medical knowledge, and the rapidly increasing facilities for its acquisition, rendered alike accessible by all the members of the profession, have brought all who are animated by a desire for its honor and usefulness, more upon a common level. The medical mind is no longer shut in and incarcerated by a blind devotion to authority. A spirit of mental independence, personal responsibility, and professional enterprise, stirs every bosom. The avenues to distinction and usefulness are alike open to all, and the pinnacle of fame and fortune holds out the most inviting attractions to the laborious aspirant. A wholesome, all-pervading spirit of emulation, leading to a riper scholarship and a more elevated standard of professional excellence, is the legitimate offspring of this equalizing process, bringing to light and establishing, in astonishingly rapid succession, new therapeutical relationships, by which the practice of our is profession becoming daily more satisfactory and successful.

I would not disguise the fact, however, that, during the last half century, the relative position of our profession and the non-medical public has undergone a marked change. Implicit confidence, amounting in some instances to blind credulity, has given place to a wide-spread skepticism as to the powers and capabilities of the healing art. This partial revolution in the public mind, which seems chiefly to have infected the ranks of the illiterate, who are incapable of taking a philosophical view of the subject, and wholly disinclined to examine its claims, has resulted from a variety of causes, somewhat obscure, but not wholly inappreciable. For this prevailing distrust as to the curative power of our art, I admit that the profession is, in part responsible; and so far, we plead guilty. The most prolific sources of skepticism however, are *extra* the profession, over which we have no control, and consequently for which we can not be held responsible.

Before attempting an enumeration of the various causes of skepticism and a demonstration of their fallacy, I will premise an assumption, the truth of which, I doubt not, will be satisfactorily vindicated in the sequel, viz: The claims of medicine the confidence of mankind, as regards its efficacy in the mitigation and cure of diseases, rest upon a basis which can not be shaken. Although it will be admitted, that, from its great abstruseness and complexity, the science of medicine is as yet, in an immature state, and consequently has not accomplished all of which it is capable; nor does it claim exemption from those frailties and miscarriages which characterize all human enterprise; still its power for good has been great; its value beyond all computation; and its promise of yet nobler achievements, in the highest degree encouraging.

Its beneficent ministrations have been described by a late writer in the following beautiful language: "Ever since the first dawn of civilization and learning, it has been the true friend of the suffering sons and daughters of men. Through its ministers and disciples it has cheered the desponding; it has lightened the load of human sorrow; it has dispelled or diminished the gloom of the sick chamber; it has plucked from the pillow of pain its thorns, and made the hard couch soft with the poppies of delicious rest; it has let the light of joy into the dark and desolate dwelling; it has rekindled the lamp of hope in the bosom of despair; it has called back the radiance of the lustreless eye, and the bloom of the fading cheek; it has sent new vigor into the failing limbs; and finally, when baffled in skill, and exhausted in all its other resources, handmaid of philosophy and religion, it has blunted the arrows of death, and rendered less rugged the precipitous, the inevitable pathway to the tomb."

In attempting, however, to vindicate the claims of medicine to the confidence of the public—exhibit the indissoluble connection subsisting between its highest state of cultivation, and the welfare of mankind, and congratulate you upon its noble achievements, I my not escape an inquiry into the degree in which it is itself responsible for the prevailing skepticism as to its competency. I have barely time briefly to advert to these causes, in which you will please indulge me, as it seemed indispensable to a lucid exposition of the subject before me.

Amongst the causes which have conspired to retard the progress, impair the usefulness, and consequently lessen public confidence in the efficacy of the medical art, may be mentioned the rage for speculation, and theorizing, which has characterized medical literature until very recently, and the almost total neglect of the only means of enriching the science, observation and experiment. It is a lamentable truth, known to every one present, that a large proportion of the ablest medical writers, both in Europe, and our own country, have written under the dominion of some favorite dogma. Instead of observing closely phenomena of nature, following her teachings, and faithfully recording facts as presented, one leading idea seems to have possessed the mind, the exclusion of all others; and the principal object, the establishment of some cherished theory, the promulgation of some favorite doctrine. It is a gratifying fact however, the one-idea age is rapidly passing away. The humoralism of Bœrhave; the nervous doctrine of Cullen; the cerebral doctrine of Clutterbuck; the physiological doctrine of Broussais; the vena-cavaism of Cooke; and the multitudes of theo-ies that have successively agitated the medical mind, ingenious and elaborate though they be, after a brief reign, have all shared the same fate, and like other remnants of antiquity—the Indian mounds of our country, or the crumbling walls, and moss-grown ruins of other lands, serve only as mementoes of past ages. "The contest between the ideal and demonstrative pholsophies has terminated in a complete triumph of the latter. A sounder, more philosophic spirit now pervades the ranks of our profession, and presides over all medical inquiry.—Still, some of the fruits of the speculative philosophy remain, to which may be attributed, in part, the immature, unsettled state of the science, and the general distrust to which we have alluded, as to the powers and capabilities of the healing art.

I hope I shall not subject myself to the charge of censoriousness, or incur the imputation of assuming to be an expounder of the principles of Ethical Philosophy, should I allude to another cause of the prevailing skepticism as to the capabilities of our art. It is a cause, insidious and elusive, but powerfully operative; and will be found to underlie, not only the general distrust which it has obtained in the public mind, but the whole compass of professional abuses and misrule, no less detrimental to the safety and welfare of society, than the respectability and usefulness of the profession. I allude to a want of a fraternising spirit in the profession, and the antagonistic position frequently assumed by its members, one towards another.

The history of man, I apprehend, affords not an instance of a more glaring misconception of true policy, than elevation and success sought by one, in disparaging and underestimating his brethren in the profession. Either from the abstruseness and complexity of the science of medicine, or a prevailing belief that its interests are separate and distinct from their own, men, otherwise intelligent, do not trouble themselves to understand the subject, although it would not be difficult to to show there is none belonging to the whole range of natural science that concerns them so much. Hence, the only competent judges of the merits of medical men, are phusicions themselves. The profession being embodied in the lives, the labors, and the condcut of its members, and each member respectively constituting an integral part of the profession, then, the character and standing of the profession, and the confidences reposed in it by the public, must depend on the acknowledged skill and ability of its members. But A disparages B, by insinuations, inuendo, or otherwise. B retorts upon A in like terms of detraction. Thus A and B both stand before the public, on the testimony of the only competent witnesses, as reprobates, and unworthy of confidence. The result of this, will be perceived, is mutal recrimination, and the disparagement of all. The extent of the inquiry accruing to the profession, by such a detraction, depends upon the proportion (God only knows how many), who are betrayed into such indiscretions. The virdict of the profession, to its claims to public confidence, and that of the non-medical public, can hardly be expected to differ. An enlightened community will not improperly conclude, that the discoverer of the motes in his brother's eye, has not his own entirely free from them. While they give credence to the presence of motes in the one case, it will be natural to suspect the existence of a beam in the other; and thus the estimate entertained of all, is lowered. When, therefore, that regard and confidence are denied us, which the efficacy of our art, and the value of our services demand, and every species of charlatanry thus finds and inlet into the various avenues of society, let us indulge a murmur, after we shall have presented an undivided front against every attack upon the character and usefulness of the profession; and evince our high appreciation of it, by exhibiting our regard for, and confidence in each other, as its exponents and representatives. Thus commending to public consideration the legitimate claims of medicine, as illustrated in the lives, the labors, and the inestimable services of a host of living cultivators and practitioners; and nobly vindicated by its progressive improvement, its onward march, its daily accessions of knowledge, its sounder, more practical philosophy, its more chaste, elevated literature, its purer, more refined ethics, and above all, its more rational accurate therapeutics, which characterize its present state, it will assuredly regain and command the regard and consideration of the public, proportioned to the brilliancy of its achievements, and the degree of its usefulness.

I propose now to call your attention, very briefly, to one or two causes of skepticism, over which our profession can exercise no control.

From a want of information amongst the masses, in regard to medical science, and a misconception of the extent of its powers of cure, extravagant expectations, and unreasonable exactions, have been indulged, admitting no apology for the circumscribed boundaries of the resources of our art. Expectation has been founded upon the assumption, that the resources of medicine are commensurate with the demand of the sick chamber. This is expecting of medicine what can be said of none of the physical sciences, a complete mastery of the subject. Medicine makes no such pretensions. Such is the complex nature of the science, and such the difficulties which surround the whole subject, that while much is known in relation to it that is practically useful, it is highly probable, that when all the elements, properties, phenomena, and relations which constitute totality in its mature state of development, are fully unfolded, it will be clearly seen that there is now, comparatively, but little known; while, over many diseases, art has acquired a gratifying control, there is still enough incompetency to humble our pride, and prompt us to still greater exertions to push our discoveries, add to our already brilliant achievements. The great intricacy of the subject, the difficulties to overcome, and the immense toil and labor requisite for rendering available even the known powers and capabilities of the art; indeed, the acknowledged imperfection of all physical science, and the circumscribed limits of human knowledge, have been lost sight of, and the expectations of the public, and the demands upon our profession, founded upon the assumptiom that science is perfect, and the resources of our art should meet all the exigencies of the sick chamber. It is no less true in morals, than in physics, that a state of excitement is, by an immutable law of nature, followed by a corresponding degree of depression. It is very easy to conceive, therefore, how unrealized expectations so extravagant, and exactions so unreasonable, should give place to doubts and apprehensions, as to the curative power of medicine. That it is appointed to man once to die, depends no less upon physical relationship, and the natural constitution of things, than the fiat of the Great Architect. That certain forms of disease, and species of injury, under the best possible remedial management, with all the lights we possess, have proven intractable, I admit; and that they will continue so, though in a greatly diminished degree, after our science shall have attained the highest possible perfection, is by no means improbable. The physician only can appreciate the complexity of the subject, and the difficulties that beset his path, in his devious search after medical truth. The subject of his inquiry is a compouud being —a rational soul, tenanting a material fabric. In consequence of this wonderful, inexplicable connection between mind and matter, we rarely find the causes of disease simple. A morbid impression upon the mind or body, acts and re-acts reciprocally, producing a complication of phenomena not easily unraveled. An infinite number of bodily organs, continually sympathizing with each other, and a countless host of moral and physical causes, daily calling these sympathies into play, it is not suprising, that an interminable list of diseases, mental and corporcal, of every grade of complication, should meet the medical philosopher at every step of his progress, and not unfrequently perplex and confound him. Nor is it surprising, that there still many pathological states for which no therapeutical relationship have been ascertained; still less marvel, that the non-medical public should fail in forming an accurate judgment, on entertaining enlightend views, in regard to the merits of medical men, or the efficacy of medical art; when, how enlightened soever upon all other subjects, it is in undeniable proof, by actual calculation, and the most accurate medical statistics, they know so little on this subject, that they are voluntarily immolated by thousands every year, on the altar of the grossest empiricism. When ever the disclosure of statistical medicine shall have revealed to the public mind the fact, that all those nations of the earth, doomed to ignorance and quackery in medicine, have greatly diminished in population, and some of them become almost extinct from the ravages of disease; and even in countries favored with scientific medicine the ratio of mortality is incomparably less in localities enjoying the counsel and advice of enlightened physicians of the regular type, than that under the regular reign of empiricism; the great importance of scientific medicine, and its indissoluble connection with the safety and welfare of society will appear so manifest, we shall not only have it honored and appreciated by the people, but fostered and protected by the State. Throw your statistical facts broadcast upon the bosom of society—let the people but see their true interest on this subject, the enlightened physician now sees it, and the regular profession will soon rise in public estimation, and empiricisn no longer curse the land with its thousands of immolated victims.

Another reason I assign, why the profession does not occupy that elevated position in society to which it is entitled, is found in the apparent contrariety in the doctrines and practices of its different members. In this, we have a striking exemplification of the incapacity of the non-medical public to render a just and fair verdict upon either the merits of medical men, or the efficacy of medical art. That there is a diversity of opinion, and a shade of difference in practice, amongst medical practitioners, is admitted. But that this difference is greatly exaggerated for want of a clear apprehension of the subject, will appear from a very slight examination. Physicians are frequently supposed to have a controversy, when in reality they are acting in perfect unison, and their apparent discrepancies are perfectly reconcilable and harmonious. For example, several physicians are summoned to the bedside of the same patient. They are alike minute in their examination of the case; and we will suppose, harmonize in their diagnose.—It is a case of venous congestion or vascular excitement, demanding depletion. A recommends the lancet. B suggests a purgative. C supposes an emetic to be indicated. D, perhaps rather more cautious and conservative, prefers reposing the case on the action of diaphoretics. Of course, the non-medical spectator, discovers in this seeming conflict, "confirmation strong as proofs of holy writ," of the truth of the proverb, "doctors will disagree;" and very safely concludes that there can be but little certainty of safety in medicine, while those who exercise it are so divergent in their views, and variant in their practices. But had he understood the subject, he would have discovered in all tnese suggestions, apparently so incongruous, the same *ultimatum.* The lancet depletes. Purgation depletes. Emesis depletes.—Diaphoresis depletes. Thus, while there is a discrepancy in mode, there is a harmony in the result. The same general indication is fulfilled by either method. I have not time nor space here to elaborate this thought, but simply remark, that when you shall have disposed of all the differences in doctrine or practice, between medical men of the regular type, which are imaginary, the objections to medicine on this ground, "will vanish into thin air," and it will be found to contain no more discordant elements than other professions. I affirm that in the cardinal principles of the science, there is a perfect argreement; and the lines of differences in practice are nearing, and will approximate, as the therapeutical relationships become more clearly ascertained, and more fully established.

I have given this rapid and very imperfect sketch of the relative positon of the medical profession, and the non-medical public, to prepare the way for a more lucid exposition of the main question before us, viz: to what extent medicine should be held responsible to the civil tribunals of the country.—*Semi-Monthly Medical News.*

[TO BE CONTINUED.]

